# A Comparative Assessment of the FDA List of 93 HPHCs in Aerosol Generated by Tobacco Heating System 2.2 versus 3R4F Reference Cigarette Smoke

**DOI:** 10.1021/acs.chemrestox.4c00544

**Published:** 2025-05-21

**Authors:** Serge Maeder, Cyril Jeannet

**Affiliations:** Philip Morris International R&D, Philip Morris Products S.A., Quai Jeanrenaud 5, Neuchâtel 2000, Switzerland

## Abstract

The US Food and Drug Administration (FDA) published an inventory of harmful and potentially harmful constituents (HPHCs), which lists 93 chemicals (FDA 93) linked to the serious health effects of tobacco use. Some of the chemical compounds in the FDA 93 list were not characterized in earlier studies due to methodological limitations at that time. Leveraging new analytical methods, the current study quantitatively assessed an expanded list of 108 HPHCs in the tobacco heating system (THS) aerosol compared with smoke from the 3R4F reference cigarette. Analyses were conducted by Labstat International ULC, an independent laboratory accredited by the Standard Council of Canada to ISO/IEC 17025:2017, on two different THS HeatStick variants (regular and menthol) together with the THS version 2.2 and the 3R4F reference cigarette smoke using a Health Canada Intense smoking regime. Of the 108 HPHCs assessed in this study, 105 were either below the limits of quantification or showed substantial reductions in THS aerosol relative to cigarette smoke (all >45%), with no increased HPHC levels in the THS aerosol relative to cigarette smoke. Aside from nicotine, anabasine, and polonium-210 (210Po) which was near the limits of detection), the average reduction in the levels of HPHCs in the aerosol of THS compared with 3R4F reference cigarette smoke was >91.6% (Regular) and >92.2% (Menthol). The results for the two THS tobacco stick variants were remarkably similar. These results confirm that the elimination of combustion in THS results in a substantial reduction of HPHCs relative to cigarette smoke.

## Graphical abstract

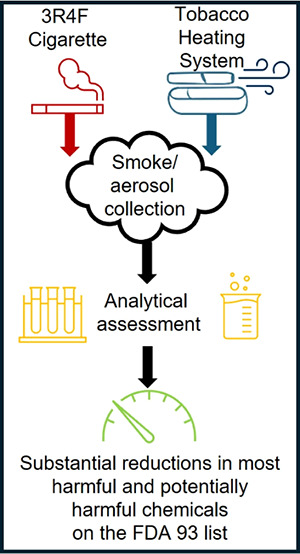

## 1 Introduction

Philip Morris Products S.A. has developed the tobacco heating system (THS), a heated tobacco product (HTP), designed to heat tobacco without initiating combustion to produce a nicotine-containing aerosol with substantially reduced numbers and levels of harmful compounds compared to those formed in cigarette smoke.

The U.S. Federal Food, Drug, and Cosmetic Act (FD&C Act) required the U.S. Food and Drug Administration (FDA) to establish a list of harmful and potentially harmful constituents (HPHCs) to be quantified in each tobacco product brand and sub-brand. In total, the 2012 established list contained 93 HPHCs (FDA 93; 119 if cresols [*o*-, *m*-, *p*-cresol] are counted as 3 and chlorinated dioxins/furans as 25).^1^ In 2019, the FDA proposed adding 19 additional HPHCs related to electronic nicotine delivery devices (ENDS) and other deemed tobacco products to the established list.^2^

Several studies have examined the HPHCs in HTPs. Forster et al. (2018)^3^ analyzed HPHCs in the aerosol from British American Tobacco’s “glo” (THP1.0) compared to 3R4F and 1R6F reference cigarettes. The THS (*IQOS* with *Essence Tobacco HeatStick*) was also analyzed for quality control, showing substantial reductions in the level of HPHCs. Reductions ranged from 84.6 to 99.9% for the 18 priority constituents identified by the US FDA.^4^ Notably, this study assessed the US FDA 93 list, excluding some compounds like polychlorinated dibenzo-p-dioxins, dibenzofurans, and radioactive isotopes, which were below detection limits.^5^ Standardized methods for measuring *N*-nitrososarcosine, coumarin, and aflatoxin B1 have not yet been developed.^3^ Additional research is needed to replicate and extend these findings.

Previously, Philip Morris International (PMI) reported the chemical composition of the aerosol emitted from the THS compared to the 3R4F reference cigarette smoke.^6^ That study quantified 59 analytes for the THS Regular variant, including 54 HPHCs listed by the public health authorities. The reduction in HPHCs was greater than 90%. This finding was confirmed later, showing a 92% average reduction in THS aerosol compared to 3R4F smoke.^7^ However, it did not include all HPHCs in the US FDA 93 list.

The limited scope of chemical assessment is also true of other studies by nonindustry researchers examining the chemistry of THS aerosol. Li et al. (2019)^8^ quantitatively compared 31 constituents of THS versus 3R4F smoke finding that the THS “delivered more than 90% fewer HPHCs, except for carbonyls, ammonia, and *N*-nitrosoanabasine, which were about 50–80% lower.” Bekki et al. (2017) found similar reductions for four tobacco-specific nitrosamines.^9^

There is a need for additional research quantifying the complete FDA 93 list of HPHCs in THS relative to that of combustible cigarette smoke. Leveraging technological advances and validating new analytical methods, the current study presents an assessment of an extended list of constituents, adding 63 HPHCs relative to the previous work.

This work is therefore aimed at verifying that the previously reported reduction of HPHCs, when tobacco is heated and not burned, is maintained over a larger selection of compounds compared with the data previously reported.

## 2 Materials and Methods

Aerosol emission and analyses were conducted by Labstat International ULC (Kitchener, ON, Canada), an ISO 17025-accredited laboratory. Labstat contracted an external laboratory for some analytes as described under Section 2.4. A summary of the methods is provided in Table S1.

### 2.1 Reference Cigarette

The 3R4F reference cigarette was obtained from the University of Kentucky (Lexington, KY, USA; https://ctrp.uky.edu/). Historically, the University of Kentucky provided reference cigarettes, differing in their design and specifications, with the aim of representing various segments of the US cigarette market. More recently, the Kentucky reference cigarette 3R4F has been widely adopted as a reference cigarette for mainstream smoke analyses and as a comparator product for cigarettes or HTPs.^10^ In this way, it provides a standard for the replication and comparison over time of the test results obtained in different laboratories.

### 2.2 Tobacco Heating System

The THS 2.2 developed by PMI is an electrically heated tobacco system that is composed of three distinct components: (1) a *HeatStick*—a novel tobacco-containing product with processed tobacco made from tobacco powder; (2) a holder, which heats the tobacco by means of an electronically controlled heating blade; and (3) a charger, which is used to recharge the holder after each use. When in use, the device generates nicotine-containing aerosol. The average temperature of the heating blade is carefully controlled and does not exceed 350 °C, which is well below the temperatures required for the combustion of tobacco to occur.

The tobacco stick was designed to be used with the THS 2.2 holder.^11^ The tobacco plug is made of reconstituted cast leaf tobacco containing various tobacco types from different origins as well as binders and glycerin as an aerosol former. To generate a visible aerosol, the tobacco is heated, evaporating the glycerin, which then recondenses to form small droplets.^6^ Two tobacco stick variants were used for aerosol characterization in this study: the THS regular and the THS menthol. These two THS *HeatSticks* contain flavor ingredients. The THS Menthol contains natural menthol applied to a cellulose acetate yarn included in the polymer-film filter and to the inner liner paper included in the *HeatStick* packet. Although the two variants of *HeatStick* are of the same design, they use different tobacco blends and flavors.

### 2.3 Aerosol Collection

The standard Health Canada Intense smoking regime (puff volume, 55 mL; puff duration, 2 s; puff interval, 30 s)^12^ was applied to both the 3R4F and the THS. For 3R4F, the ventilation blocking was 100%. Ventilation blocking was not applied to the *HeatSticks* as there are no ventilation holes in the product and, therefore, would not impact the results. For the THS aerosol samples, a total of 12 puffs were collected, corresponding to the number of puffs that can be taken during the heating duration of the THS (6 min) under the Health Canada Intense smoking regime (puff interval of 30 s). The 3R4F was smoked to a fixed butt length of 33 mm, which was normally achieved in approximately 10 puffs.

### 2.4 Quantitative Chemical Analysis

To cover the constituents listed in FDA 93, this study examined a total of 108 analytes, including nicotine. In addition to most of the HPHCs reported in a previous PMI study,^6^ this study reports results for 63 new HPHCs, including heterocyclic aromatic amines, volatile nitrosamines, chlorinated dioxins and furans, two minor alkaloids, as well as three radioactive metals, which are on the FDA 93 list of HPHCs. The polyaromatic hydrocarbons (PAHs) listed in FDA 93 (16 compounds) are reported here as well. Several points should be noted when comparing the list of compounds analyzed here to the FDA 93 list:

1 The chlorinated dioxins and furans are listed as a single entry in the FDA 93, without specifying individual compounds. These compounds were analyzed by an external laboratory (Maxxam Analytics International Corporation o/a Maxxam Anayltics 6740 Campobellon Road, Mississauga, Ontario, L5N 2L8) who analyzed their standard list of chlorinated dioxins and furans, which comprises 17 distinct compounds.
2 Cresols correspond to one entry in the FDA 93 list, whereas three isomers (orto-, meta-, and para-cresol) are reported here as individual entries.
3 Some compounds in the FDA 93 list present in tobacco, namely, aflatoxin B1, coumarin, and NSAR (*N*-nitrososarcosine), are known to be not transferred to either smoke or aerosol during combustion or heating, respectively, and so were not measured in this study.^5^

All analyses were conducted by Labstat International ULC (Kitchener, ON, Canada), except for analyses of ^210^Po, uranium, chlorinated dioxins, and furans, which were analyzed by a subcontractor (Maxxam Analytics International Corporation o/a Maxxam Anayltics 6740 Campobellon Road, Mississauga, Ontario, L5N 2L8), according to their methods and protocols. Both laboratories are ISO/IEC 17025:2017 accredited under the Testing and Calibration Laboratory Accreditation Program of the Standards Council of Canada. The list of constituents quantified as well as short summaries of the methods applied is provided in the Supporting Information.

### 2.5 Data Treatment

Values are reported as the mass of each constituent per tobacco stick. The reduction of the individual HPHCs in the THS aerosol compared to the 3R4F smoke was calculated according to

%reduction=100−100×(THSlevel/3R4Flevel)

In some cases, the results for individual constituents were below the limit of quantification (LOQ) and/or the limit of detection (LOD) for either the THS aerosol or the 3R4F smoke. In these cases, two rules were applied: (1) The value for the THS fell below the LOD/LOQ, while a value above the LOD/LOQ was reported for 3R4F; the LOD/LOQ value was used as the THS level to calculate the reduction. Therefore, the calculated reduction represents a conservative estimate, as the true reduction would be larger. In these cases, the values were thus reported as “>XX%”. (2) The values for both the THS and 3R4F fell below LOD/LOQ; no reduction calculations were performed, as both quantitative values were unknown.

## 3 Results

Levels of HPHCs for the 3R4F reference cigarette, the THS regular and THS menthol are presented in Table 1, on a per tobacco stick or per cigarette basis. The HPHCs are grouped based on their chemical classes (except for volatiles and semivolatiles, which are a heterogeneous mix of different compounds, and specific individual compounds that require dedicated methods). When the level fell below the LOQ or LOD for the THS aerosol, the corresponding LOD or LOQ value of the 3R4F smoke is indicated.

**Table 1:** Levels of HPHCs, Together with Their Standard Deviation on a per Tobacco Stick Basis, for the THS Regular and Menthol Aerosol, Compared to 3R4F Smoke (*n* = 3 Replicates)^a^ ^b^

	**Unit per **Stick****	**3R4F Cigarette**		**THS—Regular**		**THS—Menthol**		**% Reduction THS vs 3R4F**	
HPHC		Mean	SD	Mean	SD	Mean	SD	Regular	Menthol
CO	mg	30.6	1.83	<0.067	LOD	<0.067	LOD	>99.78	>99.78
Ammonia	μg	31.7	1.13	13.14	0.846	13.38	0.78	58.55	57.79
Hydrogen cyanide	μg	433	5.5	2.06	0.04	2.17	0.2	99.52	99.5
***Carbonyls***									
Formaldehyde	μg	70.2	6.17	7.1	0.607	7.68	1.234	89.89	89.06
Acetaldehyde	μg	1713	123	197.2	15.6	199.4	13.5	88.49	88.36
Acetone	μg	697	47.8	31.5	4.92	32.5	3.02	95.48	95.34
Acrolein	μg	177	15.5	9.2	0.865	9.36	0.946	94.8	94.71
Propionaldehyde	μg	125	8.97	12.2	1.16	12.4	0.93	90.24	90.08
Crotonaldehyde	μg	55.2	4.4	<3.29	LOQ	<3.29	LOQ	>94.04	>94.04
Methyl ethyl ketone	μg	184	14	7.08	0.656	7.1	0.71	96.15	96.14
***Metals***									
Mercury	ng	4.36	0.36	2.11	0.071	1.88	0.19	51.61	56.88
Cadmium	ng	99.4	4.84	<0.09	LOD	<0.28	LOQ	>99.91	>99.72
Lead	ng	<25.7	LOQ	<1.62	LOQ	<0.49	LOD	NA	NA
Chromium	ng	<11.9	LOD	<11	LOQ	<11	LOQ	NA	NA
Nickel	ng	<43.1	LOQ	<15.9	LOD	<15.9	LOD	NA	NA
Arsenic	ng	8.23	0.18	<1.2	LOQ	<1.2	LOQ	>85.42	>85.42
Selenium	ng	<4.42	LOD	<0.83	LOQ	<0.83	LOQ	NA	NA
Cobalt^c^	ng	<3.69	LOD	<3.69	LOD	<3.69	LOD	NA	NA
Beryllium^c^	pg	<11.9	LOQ	<11.9	LOQ	<11.9	LOQ	NA	NA
***Semivolatiles***									
Quinoline	μg	0.409	0.019	<0.011	LOQ	<0.011	LOQ	>97.31	>97.31
Styrene	μg	13	1.53	0.328	0.036	0.336	0.013	97.48	97.42
Nitrobenzene	μg	<0.038	LOD	<0.011	LOD	<0.011	LOD	NA	NA
Benzo[b]furan^c^	μg	0.592	0.0243	0.027	0.003	0.03	0.004	95.44	94.93
Acetamide	μg	12.3	0.354	3.28	0.116	3.21	0.067	73.33	73.9
Acrylamide	μg	4.33	0.262	1.64	0.084	1.8	0.041	62.12	58.43
***Phenolic compounds***									
Catechol	μg	98.1	7.34	12.9	0.941	12.7	0.949	86.85	87.05
Phenol	μg	14.4	0.777	0.941	0.134	0.812	0.088	93.47	94.36
*p*-Cresol	μg	6.56	0.679	<0.034	LOQ	0.04	0.003	>99.48	99.39
*m*-Cresol	μg	3.34	0.448	0.033	0.004	0.03	0.006	99.01	99.1
*o*-Cresol	μg	3.76	0.144	0.041	0.007	0.042	0.009	98.91	98.88
***PAHs***									
Naphthalene^c^	ng	1197	83.1	7.34	1.18	5.94	0.9	99.39	99.5
Benzo[a]anthracene	ng	32	2.3	2.75	0.35	2.01	0.24	91.41	93.72
Chrysene^c^	ng	41	2.8	3.9	0.4	2.9	0.32	90.49	92.93
Benzo[b]fluoranthene^c^	ng	14	0.8	1.2	0.133	0.84	0.127	91.43	94
Benzo[k]fluoranthene^c^	ng	5	0.4	0.607	0.061	<0.395	LOQ	87.86	>92.1
Benzo[a]pyrene	ng	16	0.9	1.1	0.17	0.7	0.07	93.13	95.63
Indeno[1,2,3-cd]pyrene^c^	ng	5	0.2	<0.337	LOQ	<0.337	LOQ	>93.26	>93.26
Dibenz[a,h]anthracene	ng	1	0.1	<0.124	LOD	<0.124	LOD	>87.6	>87.6
Benzo[c]phenanthrene^c^	ng	8	2.295	1.3	0.132	0.9	0.051	83.75	88.75
Cyclopenta[c,d]pyrene^c^	ng	6	0.39	2	0.26	1.1	0.15	66.67	81.67
Benzo[j]aceanthrylene^c^	ng	1.1	0.21	<0.104	LOD	<0.104	LOD	>90.55	>90.55
5-Methylchrysene^c^	ng	1.3	0.03	<0.094	LOQ	<0.094	LOQ	>92.77	>92.77
Dibenz[a,l]pyrene^c^	ng	<0.423	LOD	<0.254	LOD	<0.254	LOD	NA	NA
Dibenz[a,e]pyrene^c^	ng	<0.696	LOQ	<0.125	LOD	<0.125	LOD	NA	NA
Dibenz[a,i]pyrene^c^	ng	1.46	0.029	<0.132	LOD	<0.132	LOD	>90.96	>90.96
Dibenz[a,h]pyrene^c^	ng	<0.236	LOD	<0.141	LOD	<0.141	LOD	NA	NA
***Volatiles***									
1,3-Butadiene	μg	93	5.55	0.23	0.009	0.273	0.028	99.75	99.71
Isoprene	μg	812	11.8	1.33	0.077	1.62	0.187	99.84	99.8
Acrylonitrile	μg	22.5	1.73	<0.107	LOQ	0.112	0.039	>99.52	99.5
Benzene	μg	83.1	3.02	0.483	0.023	0.561	0.072	99.42	99.32
Toluene	μg	143	6.74	1.4	0.054	1.65	0.227	99.02	98.85
Ethylbenzene^c^	μg	14.8	0.638	0.132	0.001	0.151	0.017	99.11	98.98
Ethylene oxide	μg	21.2	2.11	0.198	0.021	0.234	0.068	99.07	98.9
Vinyl chloride	ng	128	8.1	<0.657	LOD	<0.657	LOD	>99.49	>99.49
Propylene oxide	ng	930	118	159	15.5	158	25.1	82.9	83.01
Furan^c^	μg	58.3	2.93	4.43	0.39	4.49	0.437	92.4	92.3
Vinyl acetate^c^	ng	646	44.3	60.1	1.09	66.4	5.69	90.7	89.72
Nitromethane^c^	ng	809	85.6	51.2	3.43	44.3	2	93.67	94.52
2-Nitropropane^c^	ng	36.5	6.69	8.4	0.553	6	0.261	76.99	83.56
***Aromatic amines***									
1-Aminonaphthalene	ng	18.4	0.423	<0.027	LOQ	<0.027	LOQ	>99.85	>99.85
2-Aminonaphthalene	ng	11.6	0.23	<0.012	LOQ	<0.012	LOQ	>99.9	>99.9
4-Aminobiphenyl	ng	2.81	0.238	0.008	0.0006	0.01	0.0011	99.72	99.64
2,6-Dimethylaniline^c^	ng	8.01	0.417	0.316	0.019	0.27	0.024	96.05	96.63
o-Anisidine^c^	ng	5.2	0.451	0.124	0.003	0.131	0.01	97.62	97.48
o-Toluidine	ng	105	7.39	1.08	0.05	1.08	0.089	98.97	98.97
***TSNAs***									
NNN	ng	277	39.7	15.2	1.55	9.5	1.62	94.51	96.57
NNK	ng	232	7.31	9	0.485	6.92	0.902	96.12	97.02
Caffeic acid^c^	μg	<1.19	LOD	<0.478	LOD	<0.478	LOD	NA	NA
Ethyl carbamate^c^	ng	<6.43	LOD	<1.93	LOD	<1.93	LOD	NA	NA
**Heterocyclic aromatic amines**									
IQ^c^	ng	6.73	0.757	<0.64	LOD	<0.64	LOD	>90.49	>90.49
Glu-P-2^c^	ng	<0.301	LOD	<0.12	LOD	<0.12	LOD	NA	NA
Glu-P-1^c^	ng	<0.239	LOD	<0.095	LOD	<0.095	LOD	NA	NA
PhIP^c^	ng	<0.365	LOD	<0.486	LOQ	<0.486	LOQ	NA	NA
Trp-P-2^c^	ng	6.37	0.751	<0.113	LOD	<0.113	LOD	>98.23	>98.23
AαC^c^	ng	206	4.82	1.49	0.244	1.65	0.361	99.28	99.2
Trp-P-1^c^	ng	5.2	0.872	<0.098	LOD	0.098	LOD	>98.12	>98.12
MeAaC^c^	ng	26.6	0.872	<0.385	LOQ	0.115	LOD	>98.55	>99.57
Hydrazine^c^	ng	<6.79	LOD	<2.04	LOD	2.04	LOD	NA	NA
**Volatile nitrosamines**									
NDMA^c^	ng	6.43	0.219	2.79	0.209	3.38	0.088	56.61	47.43
NEMA^c^	ng	<0.509	LOD	<0.254	LOD	<0.254	LOD	NA	NA
NDEA^c^	ng	<0.617	LOD	<0.308	LOD	<0.308	LOD	NA	NA
NPIP^c^	ng	<0.172	LOD	<0.086	LOD	<0.086	LOD	NA	NA
NPYR^c^	ng	36.8	6.41	<0.198	LOD	<0.198	LOD	>99.46	>99.46
NMOR^c^	ng	<0.55	LOD	<0.275	LOD	<0.275	LOD	NA	NA
NDELA^c^	ng	<0.085	LOD	<0.042	LOD	<0.042	LOD	NA	NA
**Chlorinated dioxins and furans**									
2,3,7,8-Tetra CDD^c^	pg	<3.9	LOD	<3.7	LOD	<3.8	LOD	NA	NA
1,2,3,7,8-Penta CDD^c^	pg	<4	LOD	<3.5	LOD	<3.9	LOD	NA	NA
1,2,3,4,7,8-Hexa CDD^c^	pg	<3.7	LOD	<3.7	LOD	<3.2	LOD	NA	NA
1,2,3,6,7,8-Hexa CDD^c^	pg	<3.7	LOD	<3.8	LOD	<3.1	LOD	NA	NA
1,2,3,7,8,9-Hexa CDD^c^	pg	<3.3	LOD	<3.4	LOD	<2.8	LOD	NA	NA
1,2,3,4,6,7,8-Hepta CDD^c^	pg	<3.5	LOD	<3.4	LOD	<20	LOQ	NA	NA
Octa CDD^c^	pg	<200	LOQ	<7.2	LOD	<200	LOQ	NA	NA
2,3,7,8-Tetra CDF^c^	pg	<3.8	LOD	<2.9	LOD	<3.1	LOD	NA	NA
1,2,3,7,8-Penta CDF^c^	pg	<3.8	LOD	<3.5	LOD	<3.3	LOD	NA	NA
2,3,4,7,8-Penta CDF^c^	pg	<4	LOD	<3.5	LOD	<3.2	LOD	NA	NA
1,2,3,4,7,8-Hexa CDF^c^	pg	<2.2	LOD	<2.4	LOD	<2.8	LOD	NA	NA
1,2,3,6,7,8-Hexa CDF^c^	pg	<2.1	LOD	<2.3	LOD	<2.7	LOD	NA	NA
2,3,4,6,7,8-Hexa CDF^c^	pg	<2.4	LOD	<2.6	LOD	<20	LOQ	NA	NA
1,2,3,7,8,9-Hexa CDF^c^	pg	<2.6	LOD	<2.9	LOD	<20	LOQ	NA	NA
1,2,3,4,6,7,8-Hepta CDF^c^	pg	<2.5	LOD	<2.7	LOD	<20	LOQ	NA	NA
1,2,3,4,7,8,9-Hepta CDF^c^	pg	<3.3	LOD	<3.6	LOD	<20	LOQ	NA	NA
Octa CDF^c^	pg	<4.4	LOD	<5.4	LOD	<200	LOQ	NA	NA
***Radio nuclides***									
Polonium-210^c^	Bq	0.0062	0.0032	<0.005	LOD	<0.005	LOD	>19.35	>19.35
Uranium-235^c^	Bq	<0.005	LOD	<0.005	LOD	<0.005	LOD	NA	NA
Uranium-238^c^	Bq	<0.005	LOD	<0.005	LOD	<0.005	LOD	NA	NA
***Alkaloids***									
Nornicotine^c^	μg	14.5	1.134	0.6	0.065	0.52	0.012	95.86	96.41
Anabasine^c^	μg	1.15	0.143	0.952	0.04	1.005	0.1	17.22	12.61

a Abbreviations: AαC, 2-amino-9H-pyrido­[2,3-b]­indole; CDD, chlorodibenzo-p-dioxin; CDF, chlorodibenzofuran; Glu-P-1, 2-amino-6-methyldipyrido­[1,2-a:3′,2’-d]­imidazole; Glu-P-2, 2-aminodipyrido­[1,2-a:3′,2’-d]­imidazole; IQ, 2-amino-3-methylimidazo­[4,5-f]­quinoline; LOD, limit of detection; LOQ, limit of quantification; MeAαC, 2-amino-3-methyl)-9H-pyrido­[2,3-b]­indole; NDEA, *N*-nitrosodiethylamine; NDELA, *N*-nitrosodiethanolamine; NDMA, *N*-nitrosodimethylamine; NEMA, *N*-nitrosomethylethylamine; NMOR, *N*-nitrosomorpholine; NPIP, *N*-nitrosopiperidine; NPYR, *N*-nitrosopyrrolidine; PhIP, 2-amino-1-methyl-6-phenylimidazo­[4,5-b]­pyridine; Trp-P-1, 3-amino-1,4-dimethyl-5H-pyrido­[4,3-b]­indole; Trp-P-2, 1-methyl-3-amino-5H-pyrido­[4,3-b]­indole; TSNAs, tobacco specific nitrosamines; NNN, Nitrosonornicotine; NNK, 4-(*N*-nitrosomethylamino)-1-(3-pyridyl)-1-butanone.

b The “<” denotes that the % reduction of THS vs 3R4F was calculated using the LOQ or LOD value for the THS.

c Denotes an HPHC that was not previously assessed in a prior PMI study^7^.

Out of the 108 HPHCs including nicotine, 40 were below the level of quantification or detection for both the 3R4F smoke and either THS aerosol variant (regular and menthol); hence, no comparison could be made. For the remaining 68 HPHCs, 21 (regular) and 20 (menthol) could not be quantified for the THS aerosol; in these cases, the LOD and/or LOQ levels of the THS aerosol were used to calculate the reduction in levels for the THS compared to the 3R4F, indicated by a “>” because reductions for constituents calculated using the LOD/LOQ represent underestimates. For all the other HPHCs, a reduction could be calculated using levels quantified for both the 3R4F and the THS (47 regular, 48 menthol).

Levels of nicotine were comparable across products (1.87 mg/stick in 3R4F smoke and 1.23 mg/stick in both THS aerosols), whereas levels of carbon monoxide were dramatically reduced (<99.7%; Table 1). Aside from nicotine, anabasine, and ^210^Po (which was near the LOD), results reveal that in all cases where a comparison could be made, the levels of HPHCs in the THS were substantially lower than those in the 3R4F (all >47%). The average reduction across all HPHCs (excluding nicotine) was >89.4% and >89.9%, respectively, for the regular and menthol THS; excluding anabasine and ^210^Po results in an average reduction of >91.6% and >92.2%, and these results are summarized in Figure 1. The findings for individual classes of compounds are presented below.

**Figure 1:**
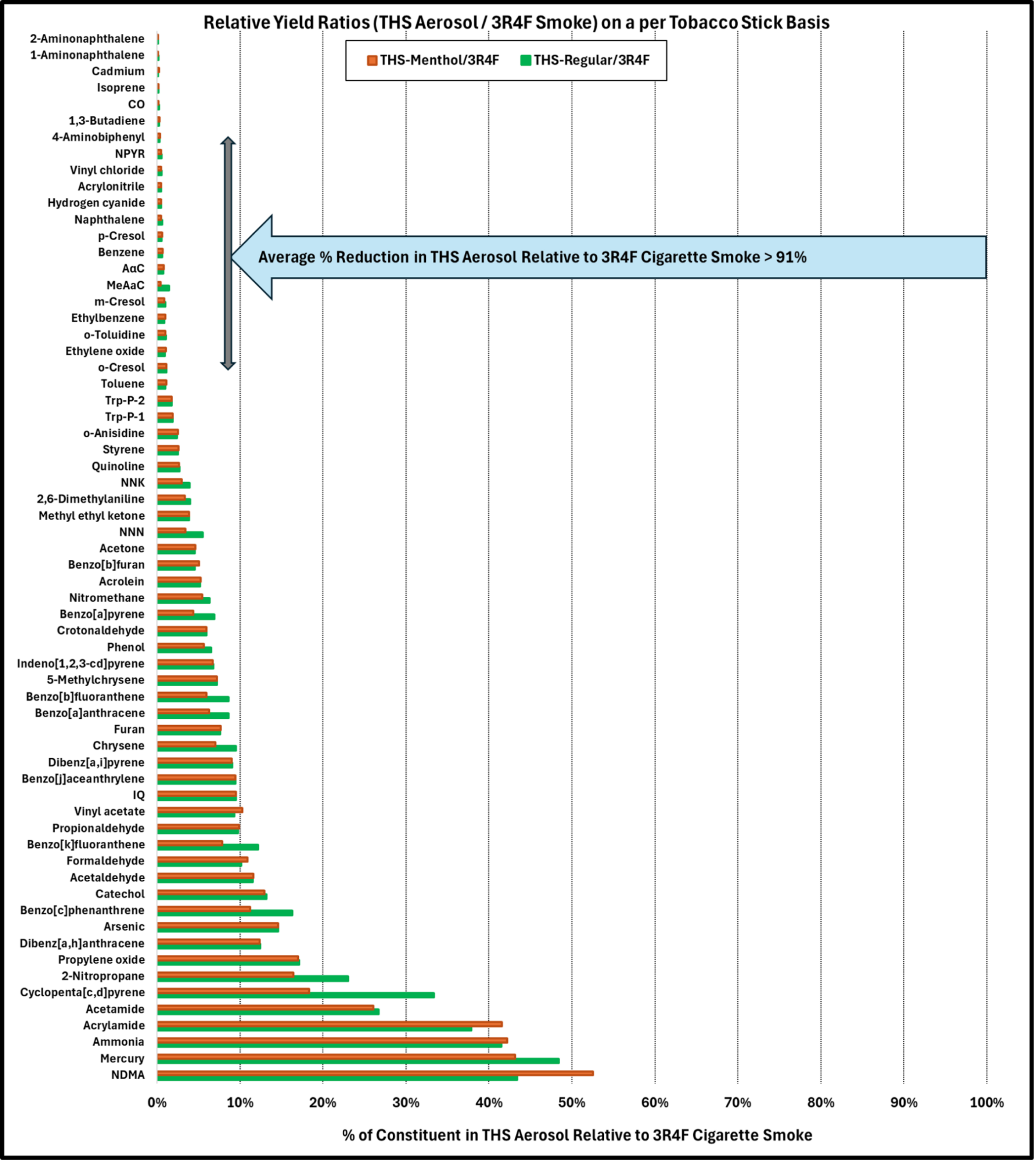
Harmful or potentially harmful constituents (HPHCs) from tobacco heating system aerosol (THS 2.2) compared to combustible cigarette smoke (3R4F reference set to 100%) on a per tobacco stick/cigarette basis under the Health Canada Intense machine-smoking regimen.

### 3.1 Nitrogenous Species

Ammonia and hydrogen cyanide were quantified for all products, each showing a substantial reduction in the THS aerosol relative to 3R4F smoke (ammonia >57%; hydrogen cyanide >99%). Ethyl carbamate and hydrazine were not previously reported. Both compounds fell below the LOD for both the 3R4F smoke and the THS aerosol.

### 3.2 Carbonyls

The seven carbonyls measured in this study (formaldehyde, acetaldehyde, acetone, acrolein, propionaldehyde, crotonaldehyde, and methyl ethyl ketone) have been previously quantified.^7^ Crotonaldehyde was below the LOQ for both THS variants. All seven carbonyls were reduced in the THS aerosol relative to the 3R4F (all >88%) with an average reduction greater than 92.5% for both the THS regular and menthol, consistent with previous reports.^6,8^

### 3.3 Metals

A total of nine metals, including seven (mercury, cadmium, lead, chromium, nickel, arsenic, and selenium) that were previously quantified^6^ and two additional metals (cobalt and beryllium), are reported here. For cobalt and beryllium, no comparison between products was made because both metals were found to be below either LOD or LOQ in 3R4F smoke and both THS aerosols. Except for mercury, which was measured in the THS aerosol at less than half the level measured in 3R4F smoke (>51% reduction), no other metal vapors were quantifiable in the THS aerosol. This is expected as the tobacco in the THS reaches a maximum temperature of 350 °C,^11^ so it remains at temperatures that would not trigger a significant transfer of metal compounds with low vapor pressures.

### 3.4 Semivolatiles

This study measured the levels of six semivolatiles (quinoline, styrene, nitrobenzene, benzo­[*b*]­furan, acetamide, and acrylamide) in THS aerosols and 3R4F smoke. Among these constituents, benzo­[*b*]­furan had not been analyzed in the previous report and was reduced by 95.4% and 95.0% in the aerosols of THS regular and menthol variants, respectively, compared to the 3R4F smoke. The other five semivolatiles were found to be reduced by similar levels to those previously reported.^6^ The average reduction for this class of constituents was >85.1% and >84.4% for the THS regular and menthol, respectively.

### 3.5 Phenolic Compounds

Among the phenolic compounds measured in this study (catechol, phenol, *p*-cresol, *m*-cresol, *o*-cresol, and caffeic acid), caffeic acid had not been analyzed in the previous report^6^ and was below LOD for all products. *p*-Cresol was below LOQ for the THS regular but quantifiable for THS menthol. The average reduction for this class of constituents was >95.5% for both THS products relative to 3R4F, which is consistent with other reports.^3,6^

### 3.6 Polycyclic Aromatic Hydrocarbons (PAH)

Among the 16 different PAHs from the FDA 93 list, three were reported previously (benzo­[*a*]­pyrene, benz­[*a*]­anthracene, and diben­[*a,h*]­anthracene),^6^ 13 additional HPHCs were detected and quantified in 3R4F smoke (in the range of 1 ng/stick to 1 μg/stick), and three were below LOD/LOQ for all products. For the THS aerosols, eight of these compounds fell below the LOQ/LOD for the regular and nine for the menthol version. Reductions relative to 3R4F were all above 66% with an average of >89.2 and >91.8 for the regular and menthol variants, respectively.

### 3.7 Volatiles

In the volatiles group, there were eight previously reported compounds (1,3-butadiene, isoprene, acrylonitrile, benzene, toluene, ethylene oxide, vinyl chloride, and propylene oxide)^7^ and five newly reported compounds (ethylbenzene, furan, nitromethane, 2-nitropropane, and vinyl acetate). All these compounds were quantifiable in cigarette smoke and showed reduced levels in the THS aerosol compared to 3R4F smoke, with an average reduction of >94.8% for regular and >95.2% for menthol.

### 3.8 Aromatic Amines

Previously, levels for four aromatic amines (1-aminonaphthalene, 2-aminonaphthalene, 4-aminobiphenyl, and *o*-toluidine) were reported.^7^ Two additional aromatic amines are reported here (2,6-dimethylaniline and *o*-anisidine), which showed substantial reductions in the THS aerosols compared to the 3R4F smoke (all >96%). Considering all six aromatic amines, the average reduction was >98.6% for both the regular and menthol THS variants.

### 3.9 Tobacco Specific Nitrosamines

Two tobacco-specific nitrosamines were quantified for all products: nitrosonornicotine and 4-(*N*-nitrosomethylamino)-1-(3-pyridyl)-1-butanone. The levels of both were dramatically reduced in THS aerosol relative to 3R4F smoke (>95%), consistent with prior reports.^3,6^

### 3.10 Heterocyclic Aromatic Amines

Among the eight newly reported compounds here, only AαC was quantified in both the 3R4F smoke and the THS aerosol. For IQ, Trp-P-2, Trp-P-1, and MeAaC, the constituents could be quantified in 3R4F smoke but were below LOD/LOQ for the THS aerosol. The other three heterocyclic aromatic amines (Glu-P-2, Glu-P-1, and PhIP) could not be quantified in neither 3R4F smoke nor the THS aerosols, so the reduction could not be calculated. Overall, the average reduction was >96.9% for both THS variants, which is likely an underestimate given that many of the THS levels were based on LOD/LOQ values.

### 3.11 Volatile Nitrosamines

For the seven newly reported volatile nitrosamines, only *N*-nitrosodimethylamine could be measured in both 3R4F smoke and the THS aerosol, with reductions in the THS aerosol of 56.6% and 47.4% for the regular and menthol variants, respectively. *N*-Nitrosopyrrolidine could be measured in 3R4F smoke but was below the LOD for the THS aerosol, with a calculated reduction of >99%. All other volatile nitrosamines were below the LOD in both the 3R4F smoke and THS aerosol.

### 3.12 Chlorinated Dioxins and Furans

For the 17 polychlorinated compounds newly analyzed in this study, all fell below the LOD or LOQ for both the 3R4F smoke and THS aerosols. The LOD/LOQ values were in the picogram range.

### 3.13 Radionuclides

Of the three newly assessed radionuclides, only ^210^Po was detected in the 3R4F smoke at a level of 6 mBk per cigarette. However, this radionuclide could not be detected in either THS aerosol. This could be because of the substantially higher boiling point of ^210^Po (962 °C) relative to the operating temperature of THS. Both uranium-238 and uranium-235 were undetected across all products.

### 3.14 Minor Alkaloids

In these analyses, while nornicotine is reduced by more than 95% in the THS aerosols compared to 3R4F smoke, anabasine is only reduced by 17.2% and 12.6% for the regular and menthol variants, respectively. The tobacco alkaloids (including nicotine) are expected to behave in a comparable manner; these compounds are present in tobacco and therefore potentially transferable into aerosols when tobacco is heated. When assessing another heat-not-burn product, the Tobacco Heating Product (THP) 1.0, Forster and colleagues (2017) also compared the alkaloid yields of this product with 3R4F smoke. The study found a greater reduction for nornicotine than for other alkaloids.^3^ This difference in transfer yields for nornicotine in HTPs in comparison with the 3R4F smoke might be explained either by differences in the efficiency of liberation of nornicotine induced by combustion in comparison with heating or by differences in the alkaloid content of the different tobacco blends. To distinguish between these possible explanations, the alkaloids were quantified in tobacco of the THS Regular variant, and the transfer yields were compared with those of the 3R4F smoke.

As shown in Table 2, the tobacco blends of the 3R4F and the THS Regular variant have a similar alkaloid composition. Therefore, the tobacco composition does not appear to be the main factor leading to the different nornicotine content of the 3R4F smoke and the THS aerosol. The transfer yield of nornicotine from the tobacco to the THS aerosol is about 10 times lower than that to 3R4F smoke, whereas the transfer yield of the other alkaloids was slightly higher for the THS aerosol. Therefore, it is likely that nornicotine requires more energy to be released from the tobacco matrix, and so a combustion process is required to obtain a more efficient transfer.

**Table 2:** Anabasine, Nicotine, and Nornicotine Content in Tobacco Filler and Mainstream Aerosol with Transfer Yields for 3R4F Cigarette and THS Regular

	**Unit**	**3R4F**	**THS Regular**
***Tobacco Filler***			
Anabasine	μg/g	140.0^a^	146.0
Nicotine	mg/g	22.5^b^	25.3
Nornicotine	μg/g	682.0^a^	637.0
***Aerosol Mainstream***			
Anabasine	μg/stk	1.15	0.95
Nicotine	mg/stk	1.87	1.23
Nornicotine	μg/stk	14.50	0.60
***Aerosol Transfer Yield***			
Anabasine	%	1.20	2.40
Nicotine	%	12.20	17.70
Nornicotine	%	3.10	0.30

a Minor alkaloid content in 3R4F tobacco obtained by Lisko et al.^13^

b Nicotine content in 3R4F tobacco obtained by Busch et al.^14^

## 4 Discussion

Leveraging the development of new analytical methods and the FDA 93 list, this study presents a more comprehensive targeted assessment of HPHCs in THS aerosol relative to that of 3R4F tobacco smoke. This study examined 63 constituents not previously examined by PMI, including heterocyclic aromatic amines, volatile nitrosamines, chlorinated dioxins and furans, two minor alkaloids, and three radioactive metals.

Overall, the results consistently demonstrate that the levels of the HPHCs in the THS aerosols were substantially reduced relative to 3R4F smoke. Among the 108 analytes, 105 were either below levels of quantification or were >45% reduced in THS aerosol relative to cigarette smoke. Among these 105 HPHCs, the average reduction was greater than 91%. Reductions for the THS Regular versus Menthol were similar (average >91.6% and >92.2%, respectively). Of the three constituents that did not show dramatic reductions, nicotine and anabasine are tobacco alkaloids, which are expected to transfer into the aerosol when tobacco is heated. The other, ^210^Po, was near the LOD for 3R4F smoke and below the LOD for the THS aerosols. The magnitude of the reduction in HPHCs presented here likely underestimates the true reductions because comparisons were made without deducting background levels. In addition, close to 30% of the estimated reductions utilize values for the LOQ/LOD for the THS aerosols, which may overestimate the true levels in the aerosol.

This substantial reduction in the levels of HPHCs in the THS aerosol relative to cigarette smoke is an important first step to suggest the potential for a reduction of HPHC exposure in THS users compared with cigarette smokers. For instance, HPHCs such as PAHs (e.g., benzo­[*a*]­pyrene), *N*-nitrosamines, heavy metals (e.g., nickel, cadmium, chromium, and arsenic), and aromatic amines have been shown to be crucial in the pathogenesis of smoking-related diseases.^15^ In this study, these compounds are greatly reduced in the THS aerosol compared to 3R4F smoke.

This study provides confirmation of the reduction in the levels of HPHCs in THS aerosol relative to cigarette smoke that were observed in prior reports.^3,6,8,9,16^ In addition, this study extends prior work by examining an expanded list of HPHCs based on the FDA 93 list and extends the findings of Cheng et al. (2022)^17^ with the assessment of THS aerosols.

One important extension was the examination of chlorinated dioxins and furans. Chlorinated dioxins were first identified in cigarette smoke by Bumb and colleagues (1980).^18^ Since tobacco contains chlorinated compounds, it was suggested that the tobacco combustion in cigarettes could generate chlorinated dioxins and dibenzofurans through pyrolytic reactions at the temperatures associated with combustion.^18^ However, in analyses of cigarette smoke, no statistically significant relationship was observed between nicotine-free dry particulate matter and chlorinated dioxin or chlorinated dibenzofuran yields.^19,20^ Since the THS does not heat tobacco above 350 °C, it does not provide favorable conditions for the formation or transfer of chlorodioxin-like compounds. Therefore, it was not surprising to observe yields below the LOD or LOQ for this product. In an analysis of 12 cigarette brands, Wilson and colleagues (2008) observed significant yields for several chlorinated dioxins and dibenzofurans.^19^ For instance, octachlorodibenzo-*p*-dioxin (octa CDD) was detected at values between 1 and 6.5 ng per cigarette. The 3R4F smoke was not analyzed by Wilson, but it was surprising not to detect octa CDD in 3R4F smoke during this study. It is possible that the relevant precursors were not present in sufficient quantities in the 3R4F blend to generate detectable levels of chlorodioxin-like compounds. The LOQ was substantially higher for octa CDD (200 pg) than for the other analytes in this group. The levels of the 17 chlorinated dioxins and furans assessed in this study were all below LOD/LOQ for the THS aerosols.

The assessment of radionuclides represents another important extension, as these were not analyzed in the previous study.^6^ They can be enriched in plant tissues when tobacco plants take them from polluted soil or air. Therefore, the concentrations of these elements in smoke or aerosols depend on the degree of air or soil pollution and the uptake rates for the individual elements. During cigarette combustion, many of the heavy metals remain in the filter and/or ash, but others are transferred into the smoke.^21^ ^210^Po is the most volatile naturally occurring radionuclide that can be detected in plants. Previously, ^210^Po was analyzed in the mainstream smoke of three cigarettes from the Portuguese market^22^ and nine cigarettes from the Japanese market.^23^ Both reported ^210^Po yields of around 1 mBk per cigarette. Uranium has also been analyzed in both cigarettes and cigarette smoke. Krivan et al. (1994)^21^ reported results for two cigarettes analyzed by four different analytical methods: atomic absorption spectrometry, instrumental neutron activation analysis, inductively coupled plasma mass spectrometry, and total reflection X-ray fluorescence. Uranium was consistently below the LODs of all of the tested methodologies. In this study, the levels of these three radionuclides in THS aerosols were all below the LOD.

The availability of 3R4F from an independent source (The University of Kentucky) allows its widespread use for tobacco research purposes, thereby providing a solid database of results for HPHC analyses and effects in biological assays supported by homogeneity of the 3R4F reference that exceeds that of most commercial products. However, using the 3R4F reference cigarette has limitations in that it was designed as a reference for an unflavored American-blend cigarette, whereas THS variants are flavored. Other work has demonstrated similar reductions in HPHCs for the THS aerosol relative to smoke from both the 3R4F and 1R6F research cigarette,^10^ and relative to smoke from cigarettes representative of the commercial market.^7^

## 5 Conclusions

Leveraging recent advances in chemical testing, this study examined THS aerosol versus smoke from a 3R4F tobacco cigarette using an expanded list of 108 HPHCs based on the FDA 93 list. Most HPHCs (105/108) were either below limits of quantification in THS aerosol or showed reductions in THS aerosol relative to cigarette smoke that exceeded 45%, with an average greater than 90%. There were no cases of increased levels of HPHCs in the THS aerosol relative to those in cigarette smoke. Further, the results for the THS tobacco stick variants were very similar, demonstrating that the elimination of combustion was by far the largest contributor to the reduction in levels of HPHCs rather than the flavor system. These results confirm that the elimination of combustion in tobacco results in a substantial reduction of HPHCs emitted into the aerosol of the THS compared to that in cigarette smoke, confirming observations reported by other PMI and independent studies. It should also be noted that not only the FDA93 analytes are reduced in THS aerosol when compared to cigarette smoke but also a large majority of the aerosol constituents not covered in this list. In a previously published study, the median reduction of all compounds detected with untargeted analytical methods was found to be ∼ 96%. In addition, a smaller number of compounds was found in THS aerosol than in cigarette smoke.^24^

## Supplementary Material



## Notes

List of analyzed harmful and potentially harmful constituents (HPHCs) and methods utilized (PDF)

## Abbreviations

CO: carbon monoxide
AαC: 2-amino-9H-pyrido­[2,3-*b*]­indole
CDD: chlorodibenzo-*p*-dioxin
CDF: chlorodibenzofuran
ENDS: electronic nicotine delivery devices
FDA: Food and Drug Administration
Glu-P-1: 2-amino-6-methyldipyrido­[1,2-*a*: 3′,2’-*d*]­imidazole
Glu-P-2: 2-aminodipyrido­[1,2-*a*: 3′,2’-*d*]­imidazole
HPHCs: harmful and potentially harmful constituents
HTP: heated tobacco product
IQ: 2-amino-3-methylimidazo­[4,5-*f*]­quinoline
LOD: limit of detection
LOQ: limit of quantification
MeAαC: 2-amino-3-methyl)-9*H*-pyrido­[2,3-*b*]­indole
NDEA: *N*-nitrosodiethylamine
NDELA: *N*-nitrosodiethanolamine
NEMA: *N*-nitrosomethylethylamine
NMOR: *N*-nitrosomorpholine
NPIP: *N*-nitrosopiperidine
PhIP: 2-amino-1-methyl-6-phenylimidazo­[4,5-*b*]­pyridine
^210^Po: polonium-210
PMI: Philip Morris International
TCTP: tobacco-containing tobacco products
THS: Tobacco Heating System
Trp-P-1: 3-amino-1,4-dimethyl-5*H*-pyrido­[4,3-*b*]­indole
Trp-P-2: 1-methyl-3-amino-5*H*-pyrido­[4,3-*b*]­indole
