# Supplementary material for: A Comparative Assessment of the FDA List of 93 HPHCs in Aerosol Generated by Tobacco Heating System 2.2 versus 3R4F Reference Cigarette Smoke
Source: Chem Res Toxicol. 2025 May 21;38(6):1037–45. doi: 10.1021/acs.chemrestox.4c00544 (PMC12175160; doi:10.1021/acs.chemrestox.4c00544)
Supplement: Supplementary file 1 [file tx4c00544_si_001.pdf]

Supporting Information

A comparative assessment of the FDA list of 93 HPHCs in aerosol generated by Tobacco Heating System 2.2 versus 3R4F reference cigarette smoke

*Serge Maeder[a] * and Cyril Jeannet[a]*

[a] Philip Morris International R&D, Philip Morris Products S.A., Quai Jeanrenaud 5, 2000 Neuchâtel, Switzerland.

* Corresponding author: Email: Serge.Maeder@pmi.com

Philip Morris International Research & Development, Philip Morris Products S.A., Quai Jeanrenaud 5, 2000 Neuchâtel, Switzerland.

Table of Content	
Table S1	Pages S1-S5

Table S1. List of Harmful and Potentially Harmful Constituents (HPHCs) Analyzed and Summary of Methods Applied.

Constituents	Health Canada / Labstat Method	Method Summary
Nicotine and carbon monoxide	T-115 / TMS-00115a	Trapping on a glass fiber pad (for nicotine) and into a vapor-phase collection bag (for CO). Analysis by GC-FID for nicotine and non-dispersive infra-red analyzer for CO.
Ammonia	T-101/ TMS-00101	Trapping on glass fiber filters followed by 2 impingers containing 0.1 N sulfuric acid. Analysis by cation exchange chromatography.
**Carbonyls:**<br>*(a)* Formaldehyde<br>*(b)* Acetaldehyde<br>*(c)* Acetone<br>*(d)* Acrolein<br>*(e)* Propionaldehyde<br>*(f)* Crotonaldehyde<br>*(g)* MEK (methyl ethyl ketone)	T-104/ TMS-00104	Trapping into an impinger containing an acidified solution of 2,4-dinitrophenylhydrazine. Analysis by reverse-phase high-performance liquid chromatography.
Hydrogen cyanide	T-107/ TMS-00107	Trapping on a glass fiber pad followed by an impinger containing a 0.1 N NaOH solution. Analysis by continuous flow colorimetric analyzer after conversion to a colored complex.
Mercury	T-108/ TMS-00108	Trapping into impingers containing an acidified potassium permanganate solution. After microwave digestion, samples were analyzed by cold vapor atomic spectroscopy.
**Metals:**<br>*(a)* Lead<br>*(b)* Cadmium<br>*(c)* Chromium<br>*(d)* Nickel<br>*(e)* Arsenic<br>*(f)* Selenium<br>*(g)* Beryllium<br>*(h)* Cobalt	T-109/ TMS-00109	Trapping with an electrostatic precipitation generator. Digestion by an acid mixture. The digestates were analyzed by inductively coupled plasma – mass spectrometry.

Constituents	Health Canada / Labstat Method	Method Summary
**Semi-volatiles:**<br>*(a)* Quinoline<br>*(b)* Styrene<br>*(c)* Benzo[b]furan<br>*(d)* Nitrobenzene<br>*(e)* Acetamide<br>*(f)* Acrylamide	T-112/<br>TMS-00112	Trapping by a glass fiber pad followed by two cryogenic traps containing methanol. Analysis by GC-MS.
**PAHs:**<br>*(a)* Naphthalene<br>*(b)* Benzo[*a*]anthracene<br>*(c)* Chrysene<br>*(d)* Benzo[*b*]fluoranthene<br>*(e)* Benzo[*k*]fluoranthene<br>*(f)* Benzo[*a*]pyrene<br>*(g)* Indeno[1,2,3-cd]pyrene<br>*(h)* Dibenz[*a,h*]anthracene<br>*(i)* Benzo[c]phenanthrene<br>*(j)* Cyclopenta[c,d]pyrene<br>*(k)* Benzo[j]aceanthrylene<br>*(l)* 5-Methylchrysene<br>*(m*) Dibenz[*a,l*]pyrene<br>*(n)* Dibenz[*a,e*]pyrene<br>*(o)* Dibenz[*a,i*]pyrene<br>*(p)* Dibenz[*a,h*]pyrene	TMS-00120	Trapping on a glass fiber pad. Analysis by GC-MS after a clean-up step.
**Alkaloids:**<br>*(a)* Nornicotine<br>*(b)* Anabasine	TMS-00127	Trapping on a glass fiber pad. Extraction with ammonium acetate followed by LC-MS/MS analysis.
**Volatiles:**<br>*(a)* 1,3-Butadiene<br>*(b)* Isoprene<br>*(c)* Acrylonitrile<br>*(d)* Benzene<br>*(e)* Toluene<br>*(f)* Ethylbenzene<br>*(g)* Ethylene oxide<br>*(h)* Vinyl chloride<br>*(i)* Propylene oxide<br>*(j)* Furan<br>*(k)* Vinyl acetate<br>*(l)* Nitromethane	TMS-00124	Trapping by a glass fiber pad followed by two cryogenic traps containing methanol. Analysis by GC-MS. The analysis of ethylene oxide requires an additional derivatization step.

Constituents	Health Canada / Labstat Method	Method Summary
2-Nitropropane	TMS-00126	Trapping with a cartridge filled with silica gel. Analysis of the eluate from the cartridge by GC.
**Aromatic amines:**<br>*(a)* *o*-Toluidine<br>*(b)* 1-Aminonaphthalene<br>*(c)* 2-Aaminonaphthalene<br>*(d)* 4-Aminobiphenyl<br>*(e)* 2,6-Dimethylaniline<br>*(f)* *o*-Anisidine	TMS-00128	Trapping on glass fiber pads. The extract was analyzed by GC-MS after a derivatization step.
**TSNAs:**<br>*(a)* *N*-nitrosonornicotine<br>*(b)* 4-(*N*-nitrosomethylamino)-1-(3-pyridyl)-1-butanone	TMS-00135	Trapping on glass fiber pads. The extract was analyzed by LC-MS/MS.
**Phenolics:**<br>*(a)* Catechol<br>*(b)* Phenol<br>*(c)* *m*-Cresol<br>*(d)* *p*-Cresol<br>*(e)* *o*-Cresol	TMS-00139	Trapping on glass fiber pads. The extract was analyzed by reversed-phase gradient liquid chromatography, using selective fluorescence detection for quantification.
Caffeic acid	TMS-00143	Trapping on glass fiber pads. The extract was analyzed by HPLC-UV detection.
Ethyl carbamate	TMS-00145	Trapping on a glass fiber pad followed by two traps containing an aqueous sulfamate buffer solution. The combined extracts were then analyzed by GC-MS-MRN after a clean-up step.
**Heterocyclic Aromatic Amines:**<br>*(a)* IQ<br>*(b)* Glu-P-2<br>*(c)* Glu-P-1<br>*(d)* PhIP<br>*(e)* Trp-P-2<br>*(f)* AαC<br>*(g)* Trp-P-1<br>*(h)* MeAαC	TMS-00146	Trapping on glass fiber pads. The extract was then analyzed by LC-MS/MS after a clean-up and concentration step.

Constituents	Health Canada / Labstat Method	Method Summary
Hydrazine	TMS-00147	Trapping on a glass fiber pad and a trap containing an aqueous buffer: methanol with 2-nitrobenzaldehyde. The extract was then analyzed by LC-MS/MS after an incubation period.
**Volatile nitrosamines**<br>N-Nitrosodiethanolamine (NDELA):<br>*(a)* NDMA<br>*(b)* NEMA<br>*(c)* NDEA<br>*(d)* NPIP<br>*(e)* NPYR<br>*(f)* NMOR<br>*(g)* NDELA	TMS-00148	Trapping into two traps containing ammonium sulfamate/sulfuric acid buffer solution followed by a glass fiber pad. The combined extract was analyzed by liquid chromatography-APCI$^+$-tandem mass spectrometry after a clean-up step.
Polonium-210	Maxxam Analytics (subcontracted laboratory by Labstat)	Trapping on a glass fiber pad. After steps of acid digestion, drying, and deposition on a silver or nickel foil, the residue was analyzed by alpha spectrometry.
Uranium	Maxxam Analytics (subcontracted laboratory by Labstat)	Quantification by neutron activation (U-238) and delayed neutron counting analysis (U-235).
Chlorinated dioxins and furans	Maxxam Analytics (subcontracted laboratory by Labstat)	Trapping on glass fiber pads. Analysis by HRGC-HRMS, after clean-up and concentration steps.

Abbreviations: AαC, 2-amino-9H-pyrido[2,3-b]indole; APCI, atmospheric pressure chemical ionization; CO, carbon monoxide; GC, gas chromatography; GC-MS-MRN, gas chromatography-mass spectrometry-Metabolite Reference Network; Glu-P-1, 2-amino-6-methyldipyrido[1,2-a:3',2'-d]imidazole; Glu-P-2, 2-aminodipyrido[1,2-a:3',2'-d]imidazole; HPLC, high-performance liquid chromatography; HRGC-HRMS, high resolution gas chromatography-high resolution mass spectrometry; IQ, 2-amino-3-methylimidazo[4,5-f]quinoline; LC-MS/MS, liquid chromatography with tandem mass spectrometry; MeAαC, 2-amino-3-methyl-9H-pyrido[2,3-b]indole; NDEA, N-nitrosodiethylamine; NDELA, N-nitrosodiethanolamine; NDMA, N-nitrosodimethylamine; NEMA, N-nitrosomethylethylamine; NMOR, N-nitrosomorpholine; NPIP, N-nitrosopiperidine; NPYR, N-nitrosopyrrolidine; PhIP, 2-amino-1-methyl-6-phenylimidazo[4,5-b]pyridine;

Constituents	Health Canada / Labstat Method	Method Summary
Trp-P-1, 3-amino-1,4-dimethyl-5H-pyrido[4,3-b]indole; Trp-P-2, 1-methyl-3-amino-5H-pyrido[4,3-b]indole; TSNAs, tobacco specific nitrosamines; UV, ultraviolet.		
